# Mitochondrial cyclophilin D ablation is associated with the activation of Akt/p70S6K pathway in the mouse kidney

**DOI:** 10.1038/s41598-017-10076-9

**Published:** 2017-09-05

**Authors:** Jelena Klawitter, Alexander Pennington, Jost Klawitter, Joshua M. Thurman, Uwe Christians

**Affiliations:** 1 Department of Anesthesiology, University of Colorado Denver, Aurora, Colorado USA; 2 Division of Renal Disease and Hypertension, University of Colorado Denver, Aurora, Colorado USA

## Abstract

The mitochondrial matrix protein cyclophilin D (CypD) is an essential component of the mitochondrial permeability transition pore (MPTP). Here we characterized the effects of CypD ablation on bioenergetics in the kidney. CypD loss triggers a metabolic shift in *Ppif−/−* male and female mouse kidneys towards glycolysis and Krebs cycle activity. The shift is accompanied by increased glucose consumption and a transcriptional upregulation of effectors of glucose metabolism in the kidney. These included activation of Akt, AMPK (only in males) and p70S6K kinases. Gender specific differences between the *Ppif−/−* male and female mouse kidneys were observed including activation of pro-surviving ERK1/2 kinase and inhibited expression of pro-apoptotic and pro-fibrotic JNK and TGFβ1 proteins in *Ppif−/−* females. They also showed the highest expression of phosphorylated-ERK1/2 and Akt S473 proteins of all four investigated animal groups. Furthermore, *Ppif−/−* females showed higher lactate concentrations and ATP/ADP-ratios in the kidney than males. These metabolic and transcriptional modifications could provide an additional level of protection to *Ppif−/−* females. In summary, loss of mitochondrial CypD results in a shift in bioenergetics and in activation of glucose-metabolism regulating Akt/AMPK/p70S6 kinase pathways that is expected to affect the capability of *Ppif−/−* mice kidneys to react to stimuli and injury.

## Introduction

In response to oxidative or other cellular stresses, mitochondrial permeability transition is accompanied by pathological and non-specific mPT pore (mPTP) opening in the inner membrane of mitochondria. mPTP can serve as a target to prevent cell death under pathological conditions such as cardiac and brain ischemia/reperfusion (I/R) injury, myocardial infarction, stroke, and diabetes^1–3^.

Cyclophilin D (CypD) is encoded by *Ppif* and is a mitochondrial peptidyl-prolyl cis-trans isomerase that has been shown to regulate the opening of the mPTP^4, 5^. Genetic deletion of CypD has been shown to reduce I/R injury, presumably by inhibiting the prolonged opening of the mPTP^4, 5^. Furthermore, the CypD inhibitor cyclosporin A (CsA) mimics the effects of CypD deletion and reduces I/R injury in animal models as well as in humans^6–9^.

The majority of the studies involving *Ppif−/−* mice have focused on its role and underlying mechanisms in the setting of myocardial and brain I/R injury. Here, CypD inhibition is beneficial by preventing necrotic cell death^5^. Recently, CypD deletion has also been shown to reduce the bone loss in aging mice^10^. On the other hand, *Ppif−/−* mice are also more susceptible to heart failure initiated by several stimuli, including physiologic exercise-induced hypertrophy^11, 12^. Mechanistically, in the heart, CypD ablation is associated with elevated levels of mitochondrial matrix Ca^2+^ that in turn leads to increased glucose oxidation relative to fatty acids. This metabolic switch limits the heart’s ability to adapt during stress^11, 13^.

In addition to the heart and brain, CypD deletion has also been shown to protect against I/R injury in the kidney^14–16^. However, much less is known yet about the effects of CypD deletion on the kidney. Ischemic kidney injury is the primary cause of acute kidney injury (AKI) in hospitalized patients^17^. We hypothesized that CypD deletion will lead to the alteration of cellular metabolism and related signaling pathways including AKT and mitogen-activated protein kinases (MAPK) in the kidney which also act as mediators of ischemic preconditioning^18, 19^.

Both clinical and experimental observations support the concept that female kidneys are less susceptible to I/R injury^20, 21^, and that women show a slower progression rate of renal disease^22^. Thus we also compared the effects of CypD deletion in kidneys from male and female mice.

## Materials and Methods

### ***In vivo*** experiments

Mice were cared for (before and during the experimental procedures) in accordance with the policies of the Institutional Animal Care and Use Committee of the University of Colorado and the National Institutes of Health *Guide for the Care and Use of Laboratory Animals*. All protocols received prior approval from the University of Colorado Institutional Animal Care and Use Committee. Wild-type (WT) B6.129SF2 mice, as well as mice null for *Ppif* on the same background (*Ppif−/−*, complete knockout), were purchased from Jackson Laboratory (Bar Harbor, ME) and were used for breeding. Age-matched homozygous KO and WT animals were used for all experiments. Animals from both types were housed in the same cage to control for environmental factors. Twelve week-old male and female mice were sacrificed, and urine (by bladder puncture) and kidney tissues were collected. For kidney collection, the renal artery was clamped, the kidney removed and immediately snap-frozen until further use.

### Tissue extraction for Western blot analysis

Frozen kidney tissues were ground to a fine powder using a mortar and pestle and solubilized in lysis buffer containing protease and phosphatase inhibitors (Pierce, Rockford, IL). The extracts were kept frozen at −80 °C for all subsequent analyses. The protein concentrations were determined using the Bradford protein assay kit (BioRad, Hercules, CA).

### Western blot analysis

For Western blots, tissue extracts were loaded onto Biorad Bis-Tris HCl Criterion gels (various percentages). Proteins were separated using a Biorad Criterion electrophoresis system operating at 120 V and then transferred from the gel to an Immobillon-P membrane (200 mA, Millipore, Billerica, MA). Membranes were incubated with the primary antibody at 4 °C overnight, after blocking with 5% milk/2% BSA in PBS-Tween buffer. Antibodies used in this study included (the majority were raised in a rabbit and to a smaller amount in a mouse): PTEN (phosphatase and tensin homolog); protein phosphatase 2 A (PP2A); AKT unmodified and phosphorylated at Ser473 and Thr308; p70S6K (unmodified and phosphorylated at T389); p38 and p42/44 MAPK (ERK1/2) unmodified and phosphorylated at Thr180/Tyr182 and Thr202/Tyr204, respectively; JNK (unmodified and phosphorylated at Thr183/Tyr185); PAK2 (p21-activated kinase) unmodified and phosphorylated at Ser20; p70 S6 kinase unmodified and phosphorylated at Thr389 and Thr421/Ser424; β-actin (source of all antibodies above Cell Signaling Technology, Danvers, MA); TGFβ1 and TGFβ3 (transforming growth factor, Santa Cruz Biotechnology, Santa Cruz, CA). After membranes were washed three times, the secondary antibody (goat-anti rabbit and horse anti-mouse antibodies conjugated to horseradish peroxidase, Cell Signaling Technology) was added. Membranes were subsequently treated with Pierce SuperSignal West Pico or Femto Solution (Pierce) following the manufacturer’s instructions. A UVP system (BioImaging Systems, Upland, CA) was used to detect the horseradish peroxidase reaction on the membrane. ImageJ software (NIH, Bethesda, MD) was used for quantitative densitometric analysis of select gel band intensities. Densitometry data were normalized to β-actin.

### Quantification of high-energy phosphate metabolites in kidney tissues

To assess the effect of CypD ablation on energy metabolism, we determined high-energy phosphate metabolite concentrations in snap-frozen kidneys using a previously described assay^23^. An average of 100 mg kidney tissue was homogenized in a mortar grinder over liquid nitrogen and extracted with 6 mL ice-cold PCA (12%) as described previously^23^. The samples were centrifuged, the liquid phase removed and neutralized to a pH of 7.0–7.3 using KOH. To separate from perchlorate salts, the neutralized samples were centrifuged again, and the supernatant lyophilized overnight. Lyophilisates were then re-dissolved in 0.5 ml water and adjusted to pH 6.5. An Agilent series 1100 HPLC (Agilent Technologies, Palo Alto, CA) coupled to an API4000 triple quadrupole mass spectrometer (AB Sciex, Concord, ON) equipped with an electrospray ionization (ESI) source was employed for quantitation of nucleotide mono-, di-, triphosphates, FAD(H_2_) and NAD(H). Energy charge was calculated as [ATP + (0.5 × ADP)]/(ATP + ADP + AMP).

### Quantification of Krebs cycle, and purine degradation metabolites in kidney tissues and urine

Citrate, succinate, glucose, α-ketoglutarate and lactate were quantified in PCA kidney tissue extracts (vide supra) and 1:20-diluted mouse urine samples (diluted with 40 nM NaOH aqueous buffer). The internal standard solution was added at a 25:1 ratio. It was prepared in 40 nM NaOH aqueous buffer and contained 200 µM d6-glucose and d4-succinate.

All samples were analyzed on an Agilent 1100 series HPLC (Agilent Technologies, Palo Alto, CA) interfaced with a positive/negative ESI API4000 tandem mass spectrometer (AB Sciex). Analytes were separated using a 150 × 3 mm Luna HILIC, 3 µm column (Phenomenex, Torrance, CA) at an HPLC solvent flow rate of 450 µL/min. The solvents were 0.1% aqueous formic acid (mobile phase A) and acetonitrile (mobile phase B). The gradient was: 0–1 min 5% acetonitrile, 1.0–3.5 min 5% acetonitrile to 15% acetonitrile, 3.5–4.5 100% acetonitrile. The column was then re-equilibrated to starting conditions (5% acetonitrile) between 4.6 and 5.5 min. The mass spectrometry parameters were (dual polarity: positive: 0–1.79 min, negative: 1.8–5.5 min): ion source gas one: 40, ion source gas two 45, source temperature 500 °C, collision gas 10, curtain gas 20, and ion source voltage of +/−4500V for positive and negative ion modes, respectively.

### Kidney histology

For hematoxylin and eosin (H&E) staining, kidney tissue samples were fixed in 10% buffered formaldehyde and embedded in paraffin, incubated for 5 minutes in Harris hematoxylin solution and for 60 seconds in eosin solution. Sections were washed with plain water, differentiated in 1% hydrochloric acid (HCl) + 50% ethanol, and stain intensity was optimized in ammonia water. Finally, sections were rinsed in 70% ethyl alcohol and dehydrated in xylene solution. *Semi quantitative scoring system* *.* The slides were scanned using an Aperio Scanscope with Spectrum Software (v. 10.0.1346.1805). The images were then analyzed using Aperio Imagescope software (v. 9.1.19.1574; APERIO Technologies, Vista, CA). Inflammatory infiltration of the kidneys was assessed by measurement of the density of nuclei using color saturation. In addition, 25 high-powered fields were examined in the tubulo-interstitium of the cortex and outer medulla of each section. The kidneys were examined for inflammation, epithelial necrosis, loss of brush border, and tubular dilatation. Twenty five glomeruli were examined for histologic changes and for vascular congestion. Their sizes as well as the average area were determined for each slide. To determine the number of glomeruli, the number of glomeruli within a region of the cortex was counted. The results are reported as the number of glomeruli corrected for the area analyzed.

### Statistical analysis

All numerical data are presented as mean ± standard deviation. One-way ANOVA with Tukey’s post hoc test was used to determine differences among groups (either between the WT and *Ppif−/−* animals within the same gender and between genders). The level of significance was set at p < 0.05 for all tests (SPSS, version 24.0, IBM/SPSS, Armonk, NY).

## Results

### Kidney histology

**Figure 1:**
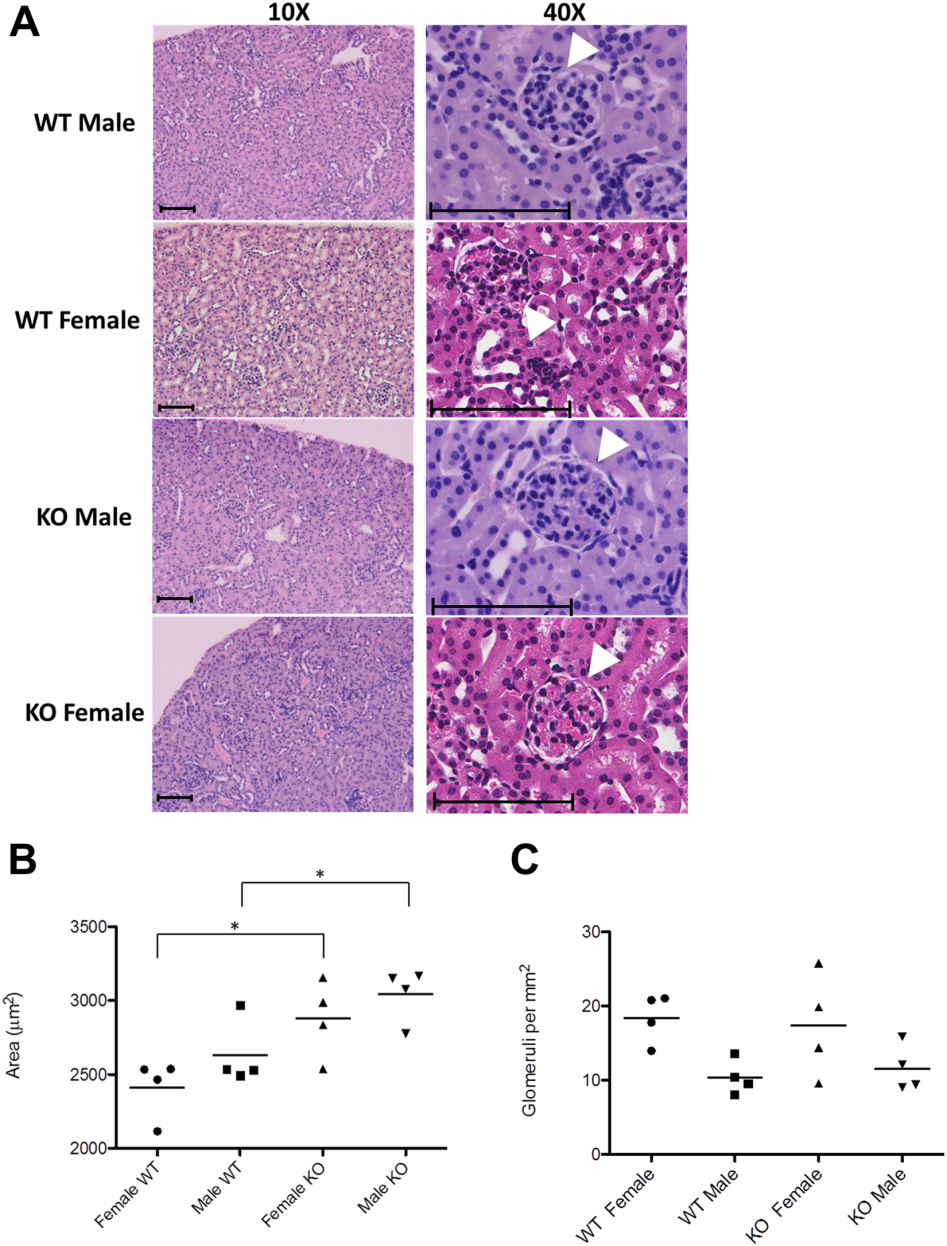
Kidney histologies of WT and *Ppif−/−* (KO) animals. (**A**) Evaluation of kidney histologies revealed a (**B**) larger area of glomeruli in *Ppif−/−* male and female mice as compared to their WT counterparts while (**C**) the number of glomeruli per mm^2^ remained the same. Arrows indicate glomeruli. Size bars indicate 200 µm (10X) and 100 µm (40x). Significance levels are: *p < 0.05 for WT versus *Ppif−/−* animals.

Comparison of kidney histologies revealed no visible differences (Fig. 1A), with the only exception that the size (area) of glomeruli were slightly enlarged in *Ppif−/−* mice as compared to their wild-type counterparts (in females and males, respectively, Fig. 1B). At the same time, the number of glomeruli remained unchanged (Fig. 1C).

### Glucose metabolism

#### Metabolite changes in the kidney

**Figure 2:**
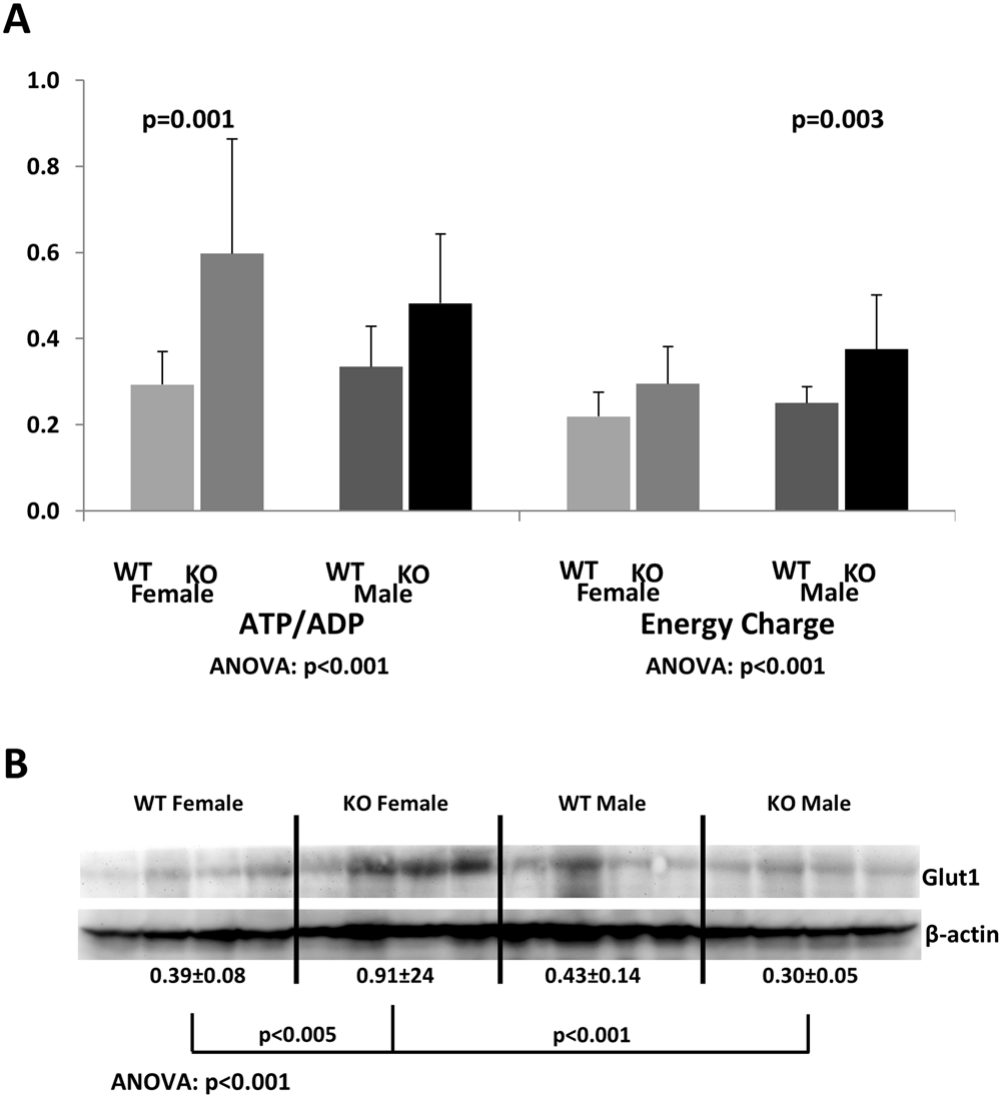
Energy state of the kidney following CypD ablation. (**A**) Comparison of ATP/ADP-ratios and energy charge between the kidneys of female and male *Ppif−/−* (KO) and wild-type (WT) mice. Data is presented as average ± SD (n = 9–14) (measured concentrations [µM] were normalized to protein [mg] prior to ratio calculations). (**B**) Expression of Glut1 glucose transporter. Data was normalized to β-actin and is presented as average ± SD (arbitrary units, n = 4). One-way ANOVA results with Tukey post-hoc significance levels are presented below. Abbreviations: ADP: adenosine diphosphate, ATP: adenosine triphosphate, AMP: adenosine monophosphate, energy charge calculated as [ATP + (0.5 × ADP)]/(ATP + ADP + AMP).

Kidneys of male *Ppif−/−* animals showed a significantly higher energy charge as compared to their wild-type counterparts (Fig. 2A). This augmented energy production was accompanied by increased glucose uptake and increased Krebs cycle activity, as suggested by the significantly higher levels of glucose, citrate and succinate in the kidney tissues of *Ppif−/−* males (Table 1). Interestingly, female *Ppif−/−* animals showed only significant increase in succinate when compared to WT females (Table 1). Their expression of glucose transporter Glut1, however, was the highest among all investigated animal groups (Fig. 2B), and so was the concentrations of the glycolysis product lactate (Table 1). *Ppif−/−* mice of both genders had higher kidney lactate concentrations as compared to their WT counterparts (Table 1).

**Table 1:** Comparison of metabolite concentrations (glucose metabolism including Krebs cycle and glycolysis) as measured in the kidneys from female and male *Ppif−/−* (KO) and wild-type (WT) mice.

	***Citrate ANOVA: p*** < ***0.001***	***α-KGD ANOVA: p*** = ***0.201***	***Succinate ANOVA: p*** = ***0.001***	***Lactate ANOVA: p*** = ***0.002***	***Glucose ANOVA: p*** = ***0.003***
WT Female	105 ± 16	48 ± 8.6	175 ± 34	1823 ± 255	1438 ± 481
KO Female	160 ± 32^#^	47 ± 14	305 ± 86^*^	2612 ± 529^**^	2156 ± 455^##^
WT Male	109 ± 30	35 ± 14	156 ± 15	1890 ± 193	1787 ± 680
KO Male	244 ± 63^***^	31 ± 18	401 ± 148^***^	2388 ± 97^*^	4498 ± 1766^***^

Data is presented as average ± SD (n = 5–6; in µM/ mg protein). One-way ANOVA significances are presented below. Tukey post hoc significance levels are as follows: *p < 0.05, **p < 0.01, ***p < 0.001 for WT versus *Ppif−/−* animals (male and female), and ^#^p < 0.05 and ^##^p < 0.01 for male versus female (*Ppif−/−* and WT, respectively). Abbreviations: α-KGD: α-ketoglutarate.

In terms of gender differences, higher intracellular kidney glucose and citrate concentrations were detected in *Ppif−/−* males as compared to corresponding females (Table 1).

#### Metabolites excretion in the urine

Only urinary citrate concentrations were higher in *Ppif−/−* males as compared to WT (Table 2). Similarly, in *Ppif−/−* females, the urinary concentrations of Krebs cycle and glycolysis intermediates and products did not differ as compared to WT females (Table 2).

**Table 2:** Comparison of metabolite as measured in the urine from female and male *Ppif−/−* (KO) and wild-type (WT) mice. Data is presented as average ± SD (n = 7–12 in µM per mM creatinine).

	***Citrate ANOVA: p*** = ***0.032***	***α-KGD ANOVA: p*** = ***0.053***	***Succinate ANOVA: p*** = ***0.496***	***Lactate ANOVA: p*** = ***0.002***	***Glucose ANOVA: p*** = ***0.058***
*WT Female*	239 ± 165	323 ± 225	67 ± 33	49 ± 26^##^	3373 ± 632
*KO Female*	353 ± 184	218 ± 128	59 ± 34	36 ± 9.0	4353 ± 2292
*WT Male*	144 ± 54	111 ± 33	42 ± 19	19 ± 9.1	2864 ± 916
*KO Male*	240 ± 105^*^	335 ± 222	63 ± 37	28 ± 13	2829 ± 800

One-way ANOVA significances are presented below. Tukey post hoc significance levels are as follows: *p < 0.05 for WT versus *Ppif−/−* animals (male and female), and ^##^p < 0.01 for male versus female (*Ppif−/−* and WT, respectively). Abbreviations: α-KGD: α-ketoglutarate.

#### Glucose Metabolism Regulation: Akt/mTOR Pathway

**Figure 3:**
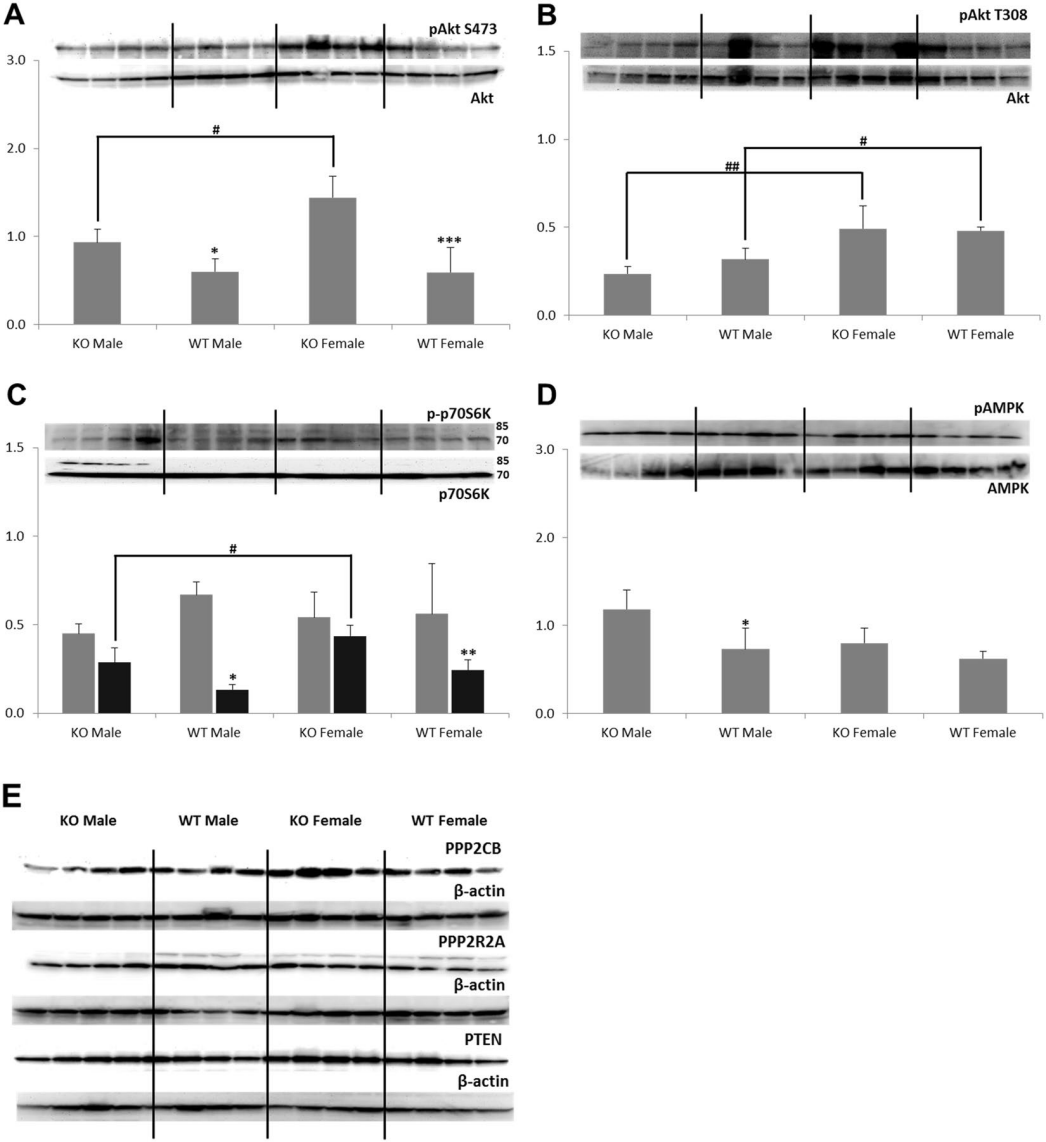
Western blot analysis of proteins involved in the regulation of glucose metabolism. (**A**) Activated Akt (presented as the phospho-Akt S473 to Akt ratio) was significantly higher in kidneys of *Ppif−/−* than in WT animals. (**B**) No change in the phospho-Akt T308 levels between *Ppif−/−* and WT animals was found. (**C**) The expression of phospho-p70S6 kinase T389 was higher in *Ppif−/−* animals and (**D**) so was expression of phospho-AMPK, however significantly only in males. (**E**) Expression of PP2A and PTEN proteins (with their respective β-actin loading controls) was similar among groups. Data is presented as average ± SD (n = 4). One-way ANOVA significances were as follows: (A) P < 0.001, (B) P < 0.001, (C) P = 0.363 and P < 0.001 for phospho-p70S6K/p70S6K 85 kD (grey bars) and 70 kDa (black bars) forms, respectively, (D) P = 0.006 and (E) P = 0.037 for PPP2CB, P = 0.009 for PP2R2A, and P = 0.281 for PTEN. Significance levels are: *p < 0.05, **p < 0.01 and ***p < 0.001 for WT versus *Ppif−/−* animals (male and female); ^#^p < 0.05 and ^##^p < 0.01 for male versus female in WT and *Ppif−/−* groups.

Activity of Akt kinase that serves as a glucose uptake and metabolism regulator was measured through its phosphorylation at two sites: S473 and T308. A significant increase in the phospho-Akt S473 to Akt ratio was observed in *Ppif−/−* animals (Fig. 3A), whereas no change in the activation of Akt at the T308 site was noted (Fig. 3B). Although no change in the phospho-Akt T308 to Akt ratio was observed, expression of its downstream target, phospho-p70S6 kinase (expressed as phospho-p70S6K/p70S6K-ratio) was significantly higher in *Ppif−/−* animals (Fig. 3C). The expression of the energy regulator AMPK was significantly higher in kidneys of male*Ppif−/−* animals (Fig. 3D). Interestingly, these increases of the phosphorylated AktS473, p70S6K and AMPK proteins, were not accompanied by changes in the expression of PPP2A phosphatases (Fig. 3E). Other than noted in heart or lung tissues^24^, but in alignment with the expression in aortic tissue^24^, naïve PTEN did not change in the kidneys of WT versus *Ppif−/−* animals (Fig. 3E).

In regards to gender differences, kidneys of *Ppif−/−* females contained higher levels of phospho-Akt S473 as compared to *Ppif−/−* males (Fig. 3A). In addition, both WT and *Ppif−/−* females expressed higher levels of phospho-Akt T308 as well as phospho-p70S6 kinase when compared to their respective male counterparts (Fig. 3B,D).

### Mitogen-activated protein kinases (MAPKs) Pathways: p38, p42/44 (ERK1/2) and JNK

**Figure 4:**
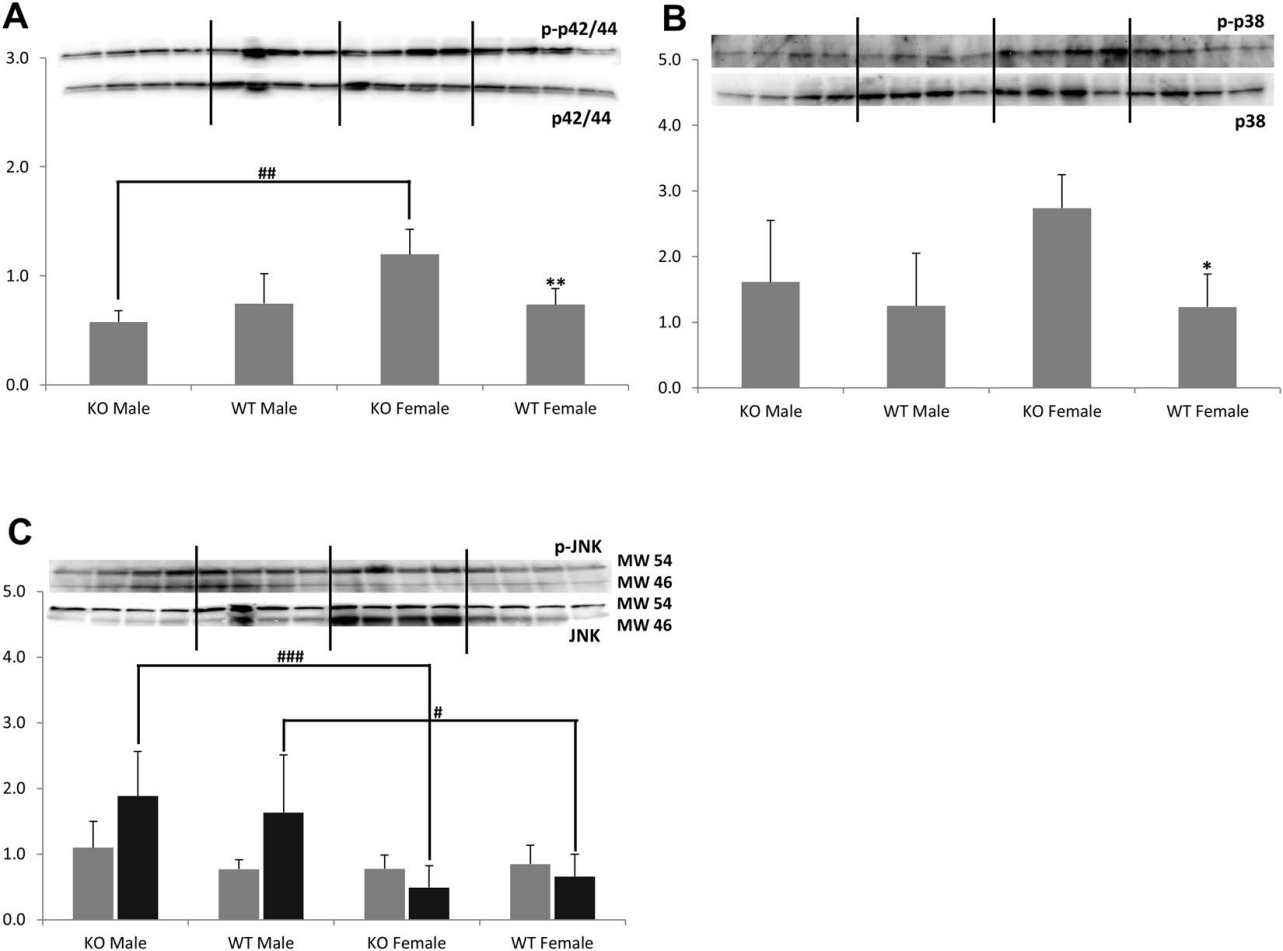
Western blot analysis of MAPK proteins. (**A**) Increased phosphorylation of p42/44 (expressed as phospho-p42/44 at Thr202/Tyr204 to p42/44-ratio) and (**B**) p38 (expressed as phospho-p38 at Thr180/Tyr182 to p38-ratio) proteins was evident in the kidneys of *Ppif−/−* females. (**C**) However, a reduction of JNK phosphorylation (phospho-JNK at Thr183/Tyr185 to JNK) was evident in both WT and *Ppif−/−* females as compared to their respective males (grey bars: band at 54 kDa, black bars: band at 46 kDa). Data is presented as average ± SD (n = 4). One-way ANOVA significances were as follows: (**A**) P = 0.005, (**B**) P = 0.035 and (**C**) P = 0.156 and P = 0.005 for pJNK/JNK 54 kD and 46 kDa forms, respectively. Significance levels are: *p < 0.05 and **p < 0.01 for WT versus *Ppif−/−* animals (male and female); ^#^p < 0.05 and ^##^p < 0.01 for male versus female in WT and *Ppif−/−* groups.

We observed no change in the phosphorylation of either p42/44 or p38 MAPKs in *Ppif−/−* males, whereas *Ppif−/−* females showed higher expression of both phosphorylated MAPK proteins (as ratios of phosphorylated to naïve protein forms) as compared to female WT controls (Fig. 4A,B, respectively). No change in the phosphorylation status of JNK MAPK was seen in *Ppif−/−* versus WT animals (phospho-JNK to JNK, Fig. 4C).

Kidneys of *Ppif−/−* females contained significantly higher levels of phospho- p42/44 MAPK, but lower levels of phospho-JNK MAPK as compared to *Ppif−/−* males (Fig. 4A,C).

### TGF-β signaling

**Figure 5:**
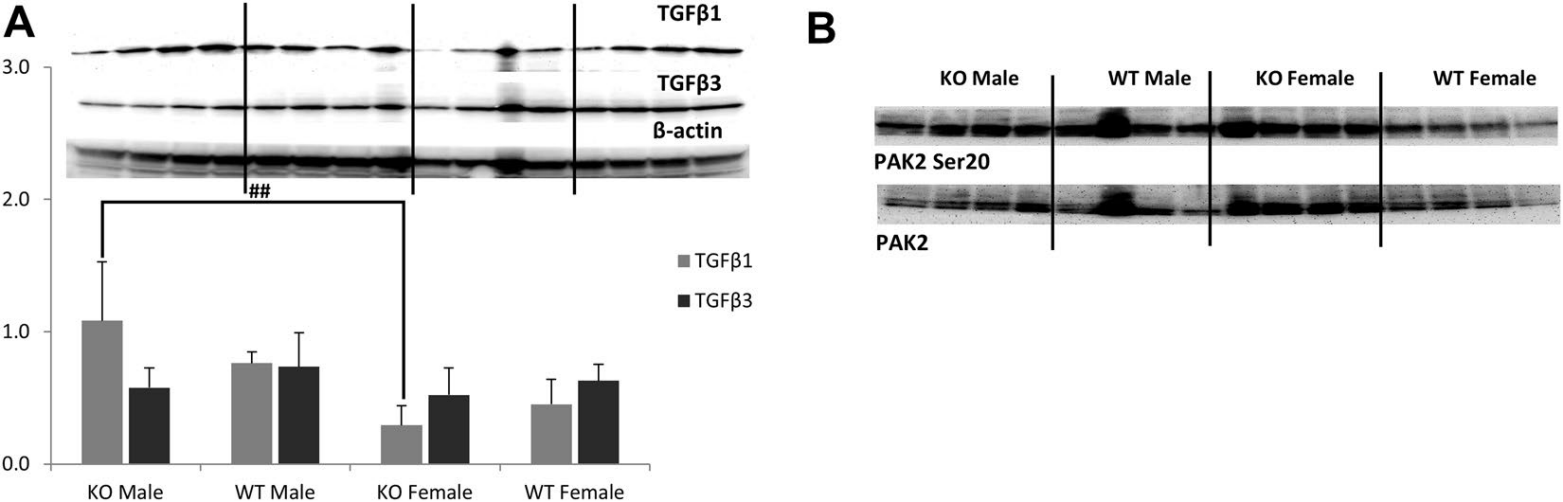
Western blot analysis of TGFβ signaling-associated proteins. (**A**) TGFβ1 was significantly higher in *Ppif−/−* males as compared to females, while no change in TGFβ3 expression was noted among the groups. (**B**) No change in the activation of PAK2 (as pPAK2 Ser20) was observed. Data is presented as average ± SD (n = 4). One-way ANOVA significances were as follows: (**A**) P = 0.004 and P = 0.469 for TGFβ1 and TGFβ3, respectively, and (**B**) P = 0.164 for pPAK Ser20/PAK2, respectively. Significance levels are: ^##^p < 0.01 for male versus female in WT and *Ppif−/−* groups.

As an interaction between high glucose concentration and the synthesis of TGFβ in renal cells^25, 26^ had been reported^24, 25^, we examined the expression of this growth factor in kidneys of *Ppif−/−* animals. Expression of TGFβ1 and TGFβ3, two proteins within the TGFβ-family associated with the development of renal fibrosis^27, 28^, remained unchanged between the *Ppif−/−* and WT animals (Fig. 5A). However, *Ppif−/−* females showed significantly lower expression of TGFβ1 as compared to the corresponding males (Fig. 5A). This observation seems interesting when considering the often slower rate of progression of renal disease in women^22^.

In addition to TGFβ, we examined the expression and activation status of the PAK2 kinase that are also involved in the development and progression of renal fibrosis. No change in the expression of unmodified or phosphorylated PAK2 protein was observed (Fig. 5B).

## Discussion

As shown in the present study, ablation of CypD is associated with reorganization of energy pathways in the kidney towards a state of higher glucose utilization.

The kidney contributes to glucose homeostasis through processes of gluconeogenesis, glucose filtration, glucose reabsorption, and glucose consumption. Krebs cycle intermediates are important substrates for renal metabolism as they account for 10–15% of oxidative metabolism in the kidney^29^. Interestingly, *in vitro* studies have shown that renal cells can be salvaged from hypoxia-reoxygenation-induced mitochondrial injury by the supplementation of Krebs cycle intermediates which mediate ATP production and prevent an energy deficit^30–32^. Furthermore, while the renal cortex uses fatty acid oxidation as the main source of energy, the renal medulla is mainly producing and taking up lactate^33^.

Morphologically, kidneys lacking CypD did not look different from those containing CypD with the only exception that CypD ablation led to enlargement of glomeruli. Metabolically, kidneys from CypD knockout mice displayed higher glucose consumption, Krebs cycle and glycolytical activity and ATP production than kidneys from WT mice. In *Ppif−/−* males, increased ATP levels were accompanied by a higher energy charge, whereas in *Ppif−/−* females concentration of AMP also increased resulting in an unchanged energy charge.

Consistent with our data, previous metabolic profiling studies suggested a switch away from fatty acid oxidation towards glycolytic and Krebs cycle metabolism in hearts, brains and livers of *Ppif−/−* animals^12, 13, 34^. In the mouse heart, genetic deletion of CypD correlated with elevated levels of mitochondrial Ca^2+^ and led to alteration in branched chain amino acid metabolism, pyruvate metabolism and the Krebs cycle^13^. However, CypD deletion did not reduce the activity of proteins involved in cellular respiration^13, 35^. Yet, in primary hepatocytes and embryonic fibroblasts, CypD loss triggered a global compensatory shift towards glycolysis, with increased glucose consumption and higher ATP production^34^. The same metabolic switch was seen in our study.

We also observed an activation of AMPK, a crucial cellular energy sensor, in male CypD-deleted mouse kidneys. In females, the degree of activation was not as pronounced and did not reach statistical significance, as was the case with the energy charge. Once activated by impaired energy metabolism, AMPK promotes ATP production by increasing the activity or expression of proteins involved in catabolism while conserving ATP by switching off biosynthetic pathways^36^. This could mean that the CypD-deleted mouse kidney is reacting to an accumulation of mitochondrial Ca^2+^ through a lower cell energy demand and redistribution of metabolic pathways, such as a reduction in fatty acid β-oxidation^13, 34, 37^, rather than stimulation of mitochondrial respiration^38, 39^. The question if this state of metabolic alteration is ultimately causing mitochondrial stress while decreasing the kidney’s metabolic reserves and rendering it more sensitive to decompensation even under subtle stress conditions, such as described for *Ppif−/−* heart^12^, remains to be investigated.

The alteration of kidney metabolism was associated with alterations in cellular signaling pathways. An upregulation of kinase cascades associated with insulin signaling including phosphorylated AMPK, Akt and mTOR was observed in kidneys of *Ppif−/−* mice. Previous studies had shown that *Ppif−/−* animals experience the expansion of insulin-producing β-cells and mild hyperinsulinemia^34^. Increased secretion of insulin could be the driving force behind the increase in Akt/mTOR and glycolytic activity, since the kidney is known to respond to insulin signaling^40, 41^. Specifically, while no change in the phosphorylation of Akt at T308 occurred, kidneys of *Ppif−/−* mice had higher levels of Akt phosphorylated at S473. Downstream, these two sites have different targets: while Akt phosphorylated at S473 is a regulating member of the glucose and lipid metabolism^42, 43^, Akt phosphorylated at T308 primarily leads to the activation of mTORC1, p70S6K, and protein synthesis^44, 45^. Therefore, the observed activation of AktS473 could be responsible for the observed glucose metabolism shift and increase in glycolysis^42, 43^. However, notwithstanding a lack of alteration in AktT308, an activation of p70S6 kinase in the kidneys of *Ppif−/−* mice was seen. This kinase has been shown to protect against I/R injury and to promote cell survival and protein production^46^.

The activation of Akt is tied to MAPK proteins including p38, p42/44 (ERK1/2) and JNK^47, 48^. These MAPKs are regulated by stress or injury not only in the kidney but also other organs. ERK promotes cell survival while JNK and p38 lead to cell death. Some studies postulate that it may be the balance between the two (ERK versus JNK) that determines the fate of the cell^49^. Interestingly, while we did not observe a change in either of these MAPKs in kidneys of *Ppif−/−* males, *Ppif−/−* females expressed higher levels of phosphorylated ERK1/2 and lower level of activated JNK kinases as compared to their wild type counterparts. *Ppif−/−* females had the highest expression of phosphorylated ERK1/2 and Akt S473 proteins among all four investigated animal groups. Furthermore, expression of the glucose transporter Glut1 was the highest in the female *Ppif−/−* kidney, which metabolically also showed the highest lactate concentration. Based on these gender differences, it may be speculated that the CypD ablation is augmenting the protective effects of estrogen in the female kidney that is known to block inflammatory and apoptotic activities^50–53^.

With the close interaction between energy demand, supply and activity of protein kinases including Akt, AMPK and mTORC1/mTORC2^44^, the observed switch in energy metabolism following CypD ablation is not surprising. The reorganization of this central controlling hub that regulates cellular functions may also be responsible for the protective effects CypD ablation has during I/R injury or stroke^5, 12, 54–56^.

**Figure 6:**
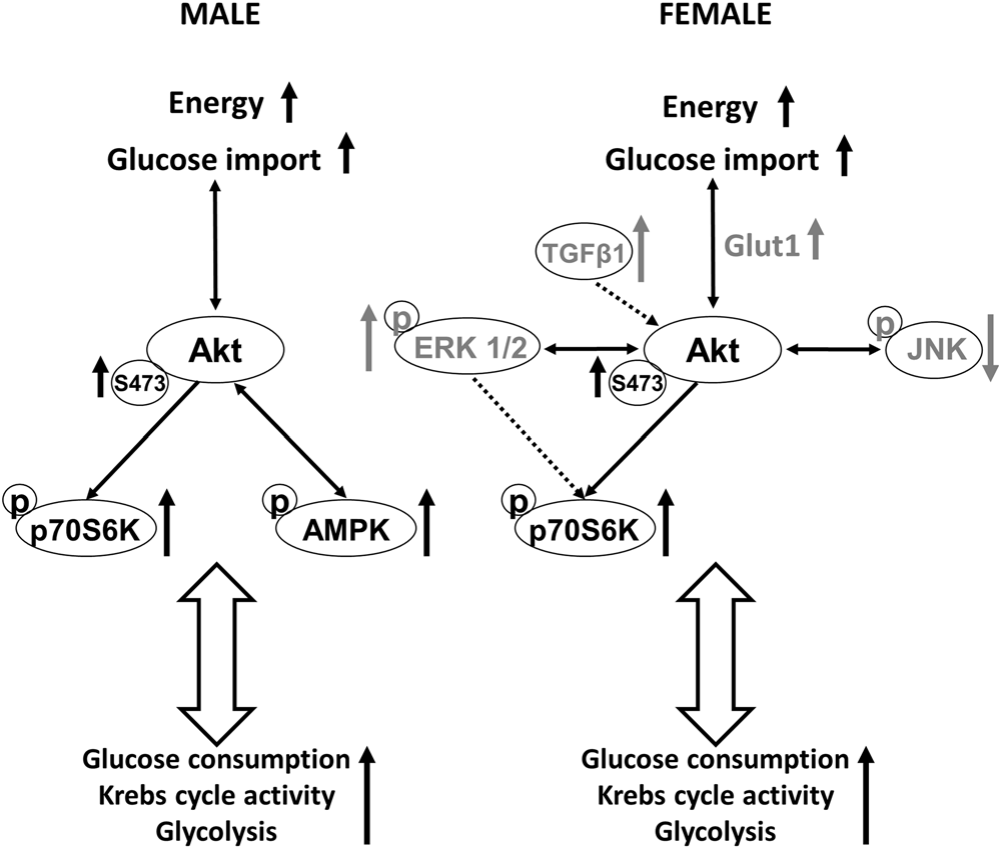
Summary of changes in cell signaling pathways in mouse kidneys as a result of CypD ablation. Increased expressions of phospho-AKT S473 and phospho-p70S6K and pAMPK (in males only) were observed in *Ppif−/−* mice. Additionally, *Ppif−/−* females (presented on the right side of the image) showed a reduced expression of TGFβ1 as well as higher expression of phosphorylated ERK1/2 and p38 proteins. ↑: increase compared to WT controls, ↓: decrease compared to WT controls. Please note that a direct link between the phosphorylation of Akt and phosphorylation of MAP kinases p38 and ERK1/2 is not fully confirmed in the here used mouse model. Please also note that, since our experiments were performed in the whole kidney, the exact contribution of each subcellular fraction to the observed changes requires further investigation.

In conclusion, it is reasonable to expect that the reorganization of cell signaling pathways and resulting metabolic changes following the loss of CypD as found in the present study (Fig. 6) leads to changes in the response of kidneys of *Ppif−/−* mice to stimuli and injury and that such responses may differ between genders.
